# Comparative Analysis of AGE and RAGE Levels in Human Somatic and Embryonic Stem Cells under H_2_O_2_-Induced Noncytotoxic Oxidative Stress Conditions

**DOI:** 10.1155/2017/4240136

**Published:** 2017-09-17

**Authors:** Maria Barandalla, Elisa Haucke, Bernd Fischer, Alexander Navarrete Santos, Silvia Colleoni, Cesare Galli, Anne Navarrete Santos, Giovanna Lazzari

**Affiliations:** ^1^Avantea srl, Laboratory of Reproductive Technologies, Cremona, Italy; ^2^Department of Anatomy and Cell Biology, Faculty of Medicine, Martin-Luther-University Halle-Wittenberg, Halle (Saale), Germany; ^3^Department of Cardiothoracic Surgery, University Hospital, Halle (Saale), Germany; ^4^Department of Medical Sciences, University of Bologna, Bologna, Italy

## Abstract

The accumulation of advanced glycation end products (AGEs) occurs in ageing and in many degenerative diseases as a final outcome of persistent oxidative stress on cells and organs. Environmental alterations taking place during early embryonic development can also lead to oxidative damage, reactive oxygen species (ROS) production, and AGE accumulation. Whether similar mechanisms act on somatic and embryonic stem cells (ESC) exposed to oxidative stress is not known; and therefore, the modelling of oxidative stress in vitro on human ESC has been the focus of this study. We compared changes in N*^ε^*-carboxymethyl-lysine (CML) advanced glycation end products and RAGE levels in hESC versus differentiated somatic cells exposed to H_2_O_2_ within the noncytotoxic range. Our data revealed that hESC accumulates CML and RAGE under oxidative stress conditions in different ways than somatic cells, being the accumulation of CML statistically significant only in somatic cells and, conversely, the RAGE increase exclusively appreciated in hESC. Then, following cardiac and neural differentiation, we observed a progressive removal of AGEs and at the same time an elevated activity of the 20S proteasome. We conclude that human ESCs constitute a unique model to study the consequence of an oxidative environment in the pluripotent cells of the embryo during the human preimplantation period.

## 1. Introduction

Aging is described as a progressive decline in the efficiency of physiological functions associated to increased susceptibility to disease and cell death [1] with the influence of genetic and environmental factors [2]. A number of studies have attempted to characterize the pathophysiology of aging and to find possibilities to counteract age-related diseases [3]. Currently, one of the most plausible and accepted mechanistic explanations of aging is the “free radical theory.” This theory states that aging and its related diseases are the consequence of reactive oxygen species- (ROS-) induced damage and of the inability to counteract these changes by endogenous antioxidant defences [4]. This unbalanced redox status is known as oxidative stress.

In general, ROS are more reactive oxygen species compared to free oxygen [5] and their effects inside the cell are determined by the subcellular source, duration, and location of these molecules [6]. In order to induce oxidative stress in a cell culture in vitro model, one of the prooxidant agents and ROS widely used is hydrogen peroxide (H_2_O_2_), which at physiological concentrations is involved in signalling mechanisms and cell metabolism. H_2_O_2_ reacts relatively slowly; however, it can also produce other ROS, which easily penetrate the cell membranes, directly damaging lipids, nucleic acids, and other macromolecules [7, 8]. H_2_O_2_ intracellular concentration is tightly controlled by various enzymatic and nonenzymatic antioxidant systems, and it is assumed to vary between 1 and 700 nM [9] depending on the cellular type and the specific system; so intracellular steady-state concentrations of H_2_O_2_ above 1 *μ*M are considered the origin of oxidative stress, inducing growth arrest and cell death [10].

Human embryonic stem cells (hESCs) have attracted huge attention for their potential applications in cell therapy, but much still remains to be explained about how their unique properties are altered by oxidative stress [11]. In preliminary studies [12], we have identified the nonscytotoxic window of exposure to H_2_O_2_ of hESCs (HUES3 and HUES7) and somatic cells (HS27 and HUVEC), being between 4 and 16 *μ*M H_2_O_2_ for Hs27 and HUES cells, and up to 32 *μ*M for HUVEC cells. In this range, we demonstrated that while no modifications of cell number and morphology occurred, a significantly increased level of ROS and lipid peroxidation was observed together with the expression of antioxidant enzymes response.

In a healthy organism during aging, the cellular proteins become increasingly damaged by ROS that induce the formation of the advanced glycation end products (AGEs) and generate ROS in a positive feedback [13, 14]. AGEs are a posttranslational modification generated by a nonenzymatic reaction, between reducing sugars and the amino groups of proteins, known as the “Maillard reaction,” which results in a change of protein structure [15]. This reaction occurs during aging and at an accelerated rate under situations such as hyperglycemia, inflammation, and/or oxidative stress conditions [16], being considered the etiology of various age-related diseases such as diabetes mellitus type 2 (DM2) [17]. Hyperglycaemia is characterized by an increase of oxidative stress biomarkers and cytokine secretion [18] and is accompanied by a decreased activity of the antioxidant systems induced by H_2_O_2_ treatment in vitro or by glucose intake in vivo [19]. High glucose environment is considered an important teratogenic factor for congenital malformations [20]; however, it is not yet clear in which way maternal hyperglycaemia and its consequent oxidative stress affects prenatal embryo development [21, 22].

Most AGE researches are performed using representative AGE compounds, like the N*^ε^*-carboxymethyl-lysine (CML) [23]. One of the major glycosylation products found in vitro is the CML modification of proteins, and it represents a general marker of both oxidative stress and long-term protein damage in diabetes and aging [24]. AGEs are able to activate intracellular cascades by binding with the AGE receptor (RAGE), which is able to transduce the molecular effects of multiple ligands [25], inducing oxidative stress and proinflammatory signalling responses [19] and mediating its pathological effects [26, 27]. Transmembrane RAGE is a cell-surface membrane protein within the immunoglobulin superfamily, constituted by three domains: a domain necessary for membrane docking, an extracellular ligand-binding domain and a cytosolic domain that is fundamental in the perpetuation of signalling events inside the cell [19]. A splice variant also exists in soluble form called sRAGE [28, 29]. The question of the functional relevance of transmembrane RAGE has become more and more popular in the last years. It is highly expressed in the early embryo at the blastocyst stage [22] and during organogenesis especially in the lung and brain [30]; then, its level decreases in nondiseased adult tissues [31], being low in neurons, endothelial cells, mononuclear phagocytes, and smooth muscle cells [30, 32]. Besides the potential role of RAGE in embryogenesis and aging processes, its physiological function is largely undefined [14]. In pathological settings such as DM, inflammation, tumours, and neurodegeneration, the RAGE expression is higher than in control animal models or human individuals [25]. AGEs and RAGE also play a central role in the pathogenesis of cardiovascular disease (CVD) [33, 34]. Diabetic micro and vascular complications are usually initiated by AGEs through the structural modification and consequent functional alteration of the proteins that are part of the extracellular matrix as well as intracellular signalling molecules. Then, engagement of RAGE with AGEs has been described that induces intracellular ROS generation and afterwards activates mitogen-activated protein kinase and nuclear factor kappa-B (NF-*κ*B) signalling, followed by production and secretion of several profibrotic and inflammatory factors [35]. Generally, RAGE is most often described to induce deleterious effects, and in previous works, the RAGE blocking showed beneficial effects in diverse diseases, suggesting RAGE as pharmaco-therapeutic target. For example, the suppression of RAGE expression by using small interference RNAs technology has been shown to contribute to the reduction of inflammation and apoptosis processes [36]. However, the modifications in receptor activity and the induced negative or positive effects of receptor-mediated AGE signalling during aging process are still to be understood.

Oxidative stress has been described critical for cell viability because the effects of mild or higher oxidation environment influences the regulation of protein production, folding changes, and proteolytic systems' adjustment. The control of protein degradation in part relies on the ubiquitin-proteasome pathway [37]. Modified proteins such as AGEs are suggested to cause cellular degeneration by forming big aggregates that not only abscond degradation but clog up the proteasomes [38] explaining why damaged and potentially proteasome-susceptible substrates accumulate with time during aging. The structure of the 26S proteasome is responsible for ATP/ubiquitin-mediated proteolysis and consists of two entities: a 20S particle that contains the proteolytic active sites and a 19S regulatory particle [39], which acts as a cap controlling the aperture of the 20S core [40]. In fact, for long time, the ubiquitin 26S/30S proteasome degradation pathway was believed to be the primary route for proteasomal degradation but is now becoming clear that although there is solid evidence that indicates that ubiquitination is involved in some forms of oxidized protein degradation [41], such proteins can also be targeted for degradation directly by the core 20S proteasome, carrying out the proteolytic activities independently of the 19S regulator [42]. Moreover, the 20S proteasome was shown to be more resistant than 26S against oxidative stress and was able to keep its activity even when the conditions induce protein damages [43]. Therefore, the substrates of the 20S proteasome are proteins that have become unfolded, partially or completely, due to mutations, aging, or oxidation [42].

Human ESCs are a valuable tool to study how the pluripotent cells in the human blastocyst stage react to an adverse environment, during the critical preimplantation window when alterations like oxidative stress can lead to future disorders, according to the Developmental Origins of Health and Disease theory (DOHaD) [44]. It has been demonstrated that in comparison with differentiated cells, murine ES cells exhibit a superior capacity to deal with external oxidative stress [45]. While the levels of telomerase activity are higher in hESC in order to protect from telomere shortening, the levels of ROS and DNA damage increase with differentiation process, suggesting that the somatic cells protect less their genome compared with their undifferentiated counterparts [46]. Interestingly, mouse ES cells contain also relatively high levels of AGEs as the early preimplantation embryo. However, the role of AGEs in ES cells and in the early mammal embryo is not understood, but it is known that they are efficiently eliminated during differentiation [47]. In murine, ESC has been demonstrated that removal of oxidatively damaged proteins is observed from the first signs of cell fate determination by an upregulation of the PA28*αβ* regulator [48].

Therefore, the objective of this study is to analyse and compare the response against oxidative stress in terms of AGE and RAGE levels between embryonic and differentiated somatic cells. We have applied a previously described noncytotoxic H_2_O_2_ treatment to generate an oxidative stress status identified by an increase of ROS [12]. Then, we have analysed the levels of AGEs and RAGE in two treated hES cells and in two treated somatic cell lines, revealing that oxidative stress affects the AGE accumulation and RAGE expression in a different way in embryonic versus differentiated cell lines. To unravel this differential response, further analysis during ES cell differentiation and in differentiated derivatives confirms that a process of efficient reduction of damaged proteins takes place in association with elevated 20S proteasomal activity.

## 2. Materials and Methods

### 2.1. Cell Culture

Human embryonic stem cells (HUES3 and HUES7 cell lines, obtained from Harvard Stem Cells Institute) [49] were first cultured on a feeder layer of mouse embryonic fibroblasts (MEFs) inactivated by mitomycin C (Sigma-Aldrich, Milan, Italy) in KO-DMEM medium (Gibco Invitrogen, Milan, Italy) supplemented with 10% serum replacement (Gibco Invitrogen, Milan, Italy), 4.3 mg/ml bovine serum albumin (BSA) (Sigma-Aldrich, Milan, Italy), 2 mM glutamine (L-alanyl-L-glutamine, Sigma-Aldrich, Milan, Italy), 1% nonessential amino acids (Gibco Invitrogen, Milan, Italy), 0.055 mM beta-mercaptoethanol (Gibco Invitrogen, Milan, Italy), 63 mg/ml penicillin, 70 mg/ml streptomycin, and 10 ng/ml bFGF (Pepro-tech, Milan, Italy). To perform the experiments, hESCs were adapted to grow in feeder-free conditions in mTeSR™1 medium (Stemcell Technologies, obtained from Voden medical instruments, Milan, Italy). The cells were passaged 1 : 4 with PBS/EDTA every 3 days, and the medium was changed daily.

Human fibroblasts (Hs27 cell line, obtained from Biobanking of Veterinary Resources, IZSLER, Brescia, Italy) were cultured in Dulbecco's Modified Eagle Medium (DMEM, high glucose, GlutaMAX™ supplement, Gibco Invitrogen, Milan, Italy), supplemented with 10% fetal bovine serum (FBS, Sigma-Aldrich, St. Louis, MO, USA). Human umbilical vein endothelial cells (HUVEC line, obtained from Biobanking of Veterinary Resources, IZSLER, Brescia, Italy) were cultured in Medium-200 supplemented with 2% low serum growth supplement (Gibco Invitrogen, Milan, Italy). Cells were passaged 1 : 3 by 0.05% trypsin/EDTA incubation at 37°C for 5 minutes (min) every 4 days.

The exposure to H_2_O_2_ started 24 hours (h) after plating, and medium was changed daily during the following 72 h, ending at day 4 after plating. Hs27 and HUVEC cells were grown in 60 mm dishes and HUES cells in 24-well plates. For immunofluorescence, detection cells were seeded on 6 mm diameter glass cover slides, and to reach the optimal cell confluence after 72 h treatment, cells were plated at different concentrations: somatic cells (Hs27 and HUVEC) were plated at 60.000 cells/ml and hESCs (HUES3 and HUES7) at 40.000 cells/ml. At the end of the treatment, cell pellets were snap-frozen for proteomic analysis and RT-qPCR or fixed in PFA (4% in PBS) for immunocytochemistry.

Replicates were performed on samples that were obtained from at least two different cell stocks that were frozen at different times and after a different number of subculture passage.

Proliferating neural precursors cells derived according to [50] were cultured on matrigel-coated dishes in DMEM-F12 medium supplemented with 2 mM glutamine, 0.6% glucose, 5 mM HEPES, 3 mM sodium bicarbonate, 60 *μ*M putrescine, 25 *μ*g/ml insulin, 100 *μ*g/ml transferrin, 20 nM progesterone, 30 nM sodium selenite, 10 ng/ml bFGF, and 2 *μ*g/ml heparin. Cells were passaged 1 : 2 twice per week.

### 2.2. In Vitro Cardiac Differentiation from hESCs (HUES7) on Feeder-Free Conditions

An established cardiac differentiation protocol from feeder-free human-induced pluripotent stem cells [51] has been utilized to differentiate hES HUES7 cell line. Embryoid body (EB) formation was initiated by harvesting HUES7 cells on feeder-free medium and transferring them to EB20 medium, which contains DMEM/F12 supplemented with 20% of fetal bovine serum albumin (BSA) (Sigma-Aldrich, Milan, Italy), 0.1 mM glutamine (Sigma-Aldrich, Milan, Italy), 0.1 mM nonessential amino acids (Gibco Invitrogen, Milan, Italy), 0.055 mM beta-mercaptoethanol (Gibco Invitrogen, Milan, Italy), 50 units/ml penicillin, 50 mg/ml streptomycin, and 50 *μ*g/ml of L-ascorbic acid (Sigma-Aldrich, Milan, Italy). After 3 days of culture, the medium was changed and at day 7 the EBs were collected for further analysis or transferred into gelatin-coated plates and cultured until the EBs were adhered to the plate tightly. Medium was changed twice per week, and contracting areas appeared after approximately 15 days. When the spontaneous contraction started, the medium was switched to EB2 medium, which was prepared as EB20 medium with 2% of FBS instead. From this moment, the medium was changed twice per week for two weeks more when cells were collected for further experiments.

### 2.3. Protein Preparation and AGE Detection by Slot Blot Analysis

For protein extraction, cell pellets from three independent replicates were dissolved separately in RIPA lysis buffer (PBS pH 7.5, 0.5% wt/vol sodium deoxycholate, 1% vol/vol NP-40, and 0.1% wt/vol SDS) with 15% of protease inhibitors (Complete Mini EDTA free, Roche, Milan, Italy) and 10% of phosphatase inhibitors (PhosSTOP, Roche, Milan, Italy), and homogenised with a syringe (26G—Terumo, Milan, Italy). After incubation on ice for 30 min, the samples were centrifuged at 14.000 rpm at 4°C for 20 min. In a new tube was collected the supernatant fraction, and the protein concentration was determined using a BCA Protein Assay kit (Pierce). Protein samples were stored at −80°C until use for slot blot or western blot analysis.

Slot blot analyses were performed on three independent replicates with 25 *μ*g protein for each. Denatured protein samples were heated for 10 min at 80° and spotted onto a nitrocellulose blotting membrane (GE Healthcare, Life Sciences, Amersham Protran) using a slot blot apparatus (TransBlot Turbo BioRad, Milan, Italy). The protein load was detected by Ponceau S staining. After blocking with Western Blocking Reagent Solution (Roche, Milan, Italy) for 1 h at room temperature (RT), the membranes were incubated with the monoclonal mouse antibody against the advanced glycation endproduct N*^ε^*-carboxymethyl-lysine (CML), the monoclonal IgG mouse antipentosidine antibody, and the monoclonal IgG antiargpyrimidine antibody, all from Biologo (Kassel, Germany), 1 : 100 in 5% milk powder and overnight at 4°C. Then, the samples were washed three times with TBS+ 1% Tween 20 (TBS-T) pH 7.5 for 5 min and incubated with a goat anti-mouse secondary antibody for 2 h (1 : 4000, Millipore, Milan, Italy) at RT. The immunoreactive signal was visualized by Luminata Forte Western HRP substrate (Millipore, Milan, Italy), and the quantification was performed measuring the intensity of the bands using ImageJ software. Protein modification rate was determined as the ratio of protein load (Ponceau) and the slot intensity by antibody reaction. The H_2_O_2_ condition values were normalized to the untreated control (CTR).

The anti-CML antibody was tested as described in Supplementary Figure S1 available online at https://doi.org/10.1155/2017/4240136, demonstrating its specificity. The production and specificity of mouse monoclonal IgG antiargpyrimidine antibody (clone 6B) was described previously [52]; and the specificity of monoclonal IgG antipentosidine (clone PEN-12) was controlled using competitive ELISA too, performed by the providing company (TansGenic Inc., Kumatomoto, Japan) as indicated in product description. Furthermore, the antipentosidine (clone PEN-12) was used by other authors for identification of pentosidine-modified proteins by Western blotting [53].

### 2.4. Western Blot Analysis

Western blot analysis was performed on three independent replicates with 25 *μ*g/lane protein loads into SDS polyacrylamide gel (Mini-Protean TGX, Stain-free gels 10%, BioRad, Milan, Italy) and transferred with a Trans Blot Turbo machine (BioRad, Milan, Italy) to a nitrocellulose blotting membrane (GE Healthcare, Life Sciences, Amersham Protran) followed by immunoblotting. Membranes were blocked with Western Blocking Reagent Solution (Roche, Milan, Italy) for 1 h at RT. The membranes were then incubated overnight at 4°C with the goat polyclonal antibody against C-terminus of RAGE (1 : 100, sc-8229, Santa Cruz Biotechnologies Inc., Milan, Italy). Samples were washed three times with TBS-T for 5 min and then incubated with an anti-goat IgG secondary antibody for 2 h (1 : 250, Millipore, Milan) at RT.

The immunoreactive signal was visualized by Luminata Forte Western HRP substrate (Millipore, Milan, Italy). Quantification was performed measuring the intensity of the bands using ImageJ software. Membranes were stripped in harsh solution (10% SDS, Tris HCl 0.5 M pH 6.8 and 0.7% *β*-mercaptoethanol in H_2_O) during 50 min at 50°C, washed three times with TBS-T for 5 min and left blocking overnight at 4°C. The membranes were then incubated with the mouse monoclonal antibody against *β*-actin (1 : 35.000, ab6276, abcam) for 2 h at RT. After that, membranes were washed three times with TBS-T for 5 min and then incubated with an anti-mouse IgG secondary antibody for 1 h (1 : 4000, Millipore, Milan) at RT. The same membranes were probed for both target protein and loading control. Protein modification rate was calculated as the ratio between *β*-actin signal and the RAGE intensity by antibody reaction. The H_2_O_2_ condition values were normalized to the untreated control (CTR).

### 2.5. 20S Proteasome Activity Assay

Cells from three independent experiments were lysed separately using RIPA lysis buffer (PBS pH 7.5, 1% vol/vol NP-40, 0.5% wt/vol sodium deoxycholate, and 0.1% wt/vol SDS) and homogenised with a syringe (26G—Terumo, Milan, Italy). After incubation on ice for 30 min, the samples were centrifuged at 14.000 rpm at 4°C for 20 min. In a new tube, the supernatant fraction was collected and the protein concentration was determined using a Pierce BCA Protein Assay kit (Thermo Scientific, Milan, Italy). The proteasomal activity was measured using the 20S Proteasome Activity Assay kit according to the manufacturer's instructions (APT280, Chemicon, Millipore). Briefly, the lysates (25 *μ*g of each protein sample) were incubated with the fluorogenic substrate LLVY-AMC (a fluorophore 7-amino-4-methylcoumarin [AMC] bound with LLVY peptide) in a 96-well fluorometer plate at 37°C for 1.5 h. LLVY is a substrate identified and cleaved by the 20S proteasome. After LLVY chymotryptic cleavage by the 20S proteasome, AMC is released and emits fluorescence that can be read in a fluorometer by using a 380/460 nm excitation and emission filters, in this case in a Tecan Infinite F200 PRO microplate reader (Tecan Italia srl, Cernusco sul Naviglio, Italy). Proteasome activity is expressed as relative fluorescent units (RFU) of the inhibited fraction of AMC signal with Lactacystin 20S proteasome inhibitor of the *β*5 subunit [54] provided in the kit.

### 2.6. RNA Isolation, cDNA Synthesis, and qPCR

RNA was extracted from control and treated cells, from three different independent replicates, using the RNeasy Mini Kit (Qiagen, Milan, Italy) following the manufacturer's instructions. Immediately after extraction, the reverse transcription reaction was carried out with iScriptTM cDNA Synthesis Kit (Bio-Rad, Milan, Italy) following the manufacturer's instructions. Tubes were first incubated at 25°C for 5 min and then at 42°C for 30 min to allow the reverse transcription of mRNA, followed by 85°C for 5 min to denature the enzyme.

The amount of RAGE and cardiac validation gene transcripts was determined by real-time quantitative PCR (RT-qPCR). Three-control cDNA replicates and three replicates of each treatment condition (4, 8, 16, and 32 *μ*M of H_2_O_2_) were conducted for all genes of interest. PCR was performed with the PCR mix iTaqTM Universal SYBR Green Supermix (Bio-Rad, Milan, Italy) containing the specific primers (Supplementary Table T1) in a MyiQ Real-Time PCR Detection System (Bio-Rad, Milan, Italy). Data were analysed with the iQ Optical System Software (Bio-Rad) by the ddCt method. 18S was used as housekeeping reference gene. Expression amounts of the examined genes were normalized to the untreated control, CTR.

### 2.7. Immunofluorescence of CML and RAGE

In order to localize the CML and RAGE, cells grown at glass cover slides were washed once with PBS and fixed in 4% paraformaldehyde (VWR, Milan, Italy) for 30 min at RT. Then, they were permeabilized by incubation in 0.5% Triton (Sigma, Milan, Italy) in PBS for 15 min at RT and blocked in 10% chicken serum (Sigma, Milan, Italy) in PBS for 1 hour at RT. After that, cells were incubated 2 h at RT with the monoclonal mouse antibody against CML (1 : 100, Biologo, Kassel, Germany) and goat polyclonal antibody against RAGE (1 : 100, sc-8229, Santa Cruz Biotechnologies Inc, Milan, Italy). Following incubation, cells were washed three times and incubated with secondary antibodies 1 : 200 Fitc anti-mouse and 1 : 150 Texas-Red anti-goat (Jackson ImmunoResearch, Milan, Italy) for 1.5 hours in the dark at RT. Finally, cells were incubated with 5 *μ*g/ml Hoechst 33342 (Sigma, Milan, Italy) for 15 min in the dark at RT and washed three times in PBS and mounted with Citifluor (Citifluor Ltd., London, UK). Slides were observed by fluorescence microscopy (Nikon Eclipse 80i). Negative controls were performed with omission of the primary antibody before secondary antibody addition.

### 2.8. Statistical Analysis

All values are expressed as mean ± standard deviation (SD) and were obtained from three separate replicates. Statistical analysis was assessed by one-way ANOVA and Tukey's post hoc test for multiple comparisons. All statistical calculations were computed using GraphPad PRISM software version 6 (GraphPad Software, San Diego, CA), ∗ indicates significant differences, *p* val ≤0.05; ^∗∗^*p* val ≤0.01; ^∗∗∗^*p* val ≤0.001, and ^∗∗∗∗^*p* val ≤0.0001.

## 3. Results

### 3.1. Detection of Advanced Glycation End-Product Proteins in Treated hES and Somatic Cell Lines

**Figure 1:**
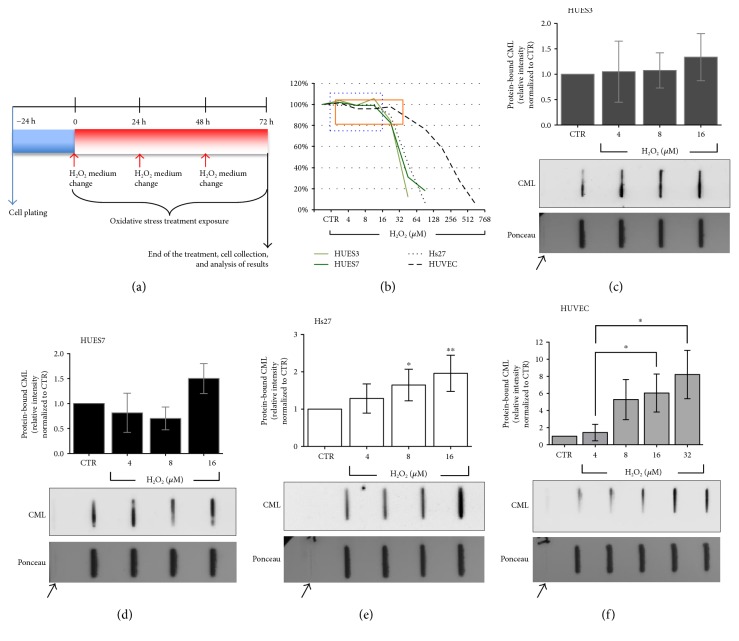
Hydrogen peroxide treatment diagram, dose-response curves following hydrogen peroxide (H_2_O_2_) 72 h exposure and relative amount of CML in protein extracts of hES and somatic cells after 72 h of noncytotoxic H_2_O_2_ oxidative stress treatment. (a) Hydrogen peroxide treatment diagram. Representative scheme for 72 h H_2_O_2_ oxidative stress treatment. At the end of the protocol, 3 days after the initiation of chemical exposure, the viability, gene expression, and protein levels were evaluated in exposed versus not exposed control (CTR) cells. (b) Dose-response curves, modified from Barandalla et al. [12]. HUES3, HUES7, Hs27, and HUVEC were exposed to increasing concentrations of hydrogen peroxide for 72 hours, and cell viability was determined by AlamarBlue reagent. *Blue dotted square* highlights noncytotoxic range for HUES3, HUES7, and Hs27 cells, between 4 and 16 *μ*M. *Orange solid square* highlights noncytotoxic range for HUVEC, between 4 and 32 *μ*M. Data (means ± SD, 3 separate replicates per H_2_O_2_ experimental condition) are expressed as percentages of cell viability relative to the respective CTR, untreated control cells. (c–f) Relative amount of CML in protein extracts of hES and somatic cells after 72 h of noncytotoxic H_2_O_2_ oxidative stress treatment. Relative amount of protein-bound N*^ε^*-carboxymethyl-lysine (CML) in the H_2_O_2_-treated cells normalized with the nontreated cells. The CML amount is related to the protein load (Ponceau S staining). A representative slot blot is shown for each cell line. PBS was used as a negative control (arrows). The quantification was performed by slot blot analysis: mean ± SD, 3 separate replicates per H_2_O_2_ experimental condition. Statistically significant differences between groups are indicated as follows: ^∗^*P* ≤ 0.05 and ^∗∗^*P* ≤ 0.01 as established by one-way ANOVA and Tukey's post hoc test.

In a previous work [12], we developed a novel in vitro model to analyse the effects of oxidative stress and the antioxidant response against reactive oxygen species (ROS) on embryonic stem cells in comparison with somatic cells, demonstrating that the nonlethal doses of H_2_O_2_ resulted in an increase in oxidative stress in treated cells. To evaluate the nominal concentration-effect relationship for the cytotoxic action of H_2_O_2_, human somatic cells (Hs27 and HUVEC) and embryonic stem (HUES3 and HUES7) cell lines were exposed to rising concentrations of H_2_O_2_ between 4 and 768 *μ*M during 72 h and cell viability was analysed by AlamarBlue® reduction and normalized to the nontreated control samples of each cell line (Figure 1(a)).

To determine the AGE status of the cells under oxidative stress conditions, we quantified specific protein-bound AGEs by slot blot analysis in three independent experiments of treated HUES3 and Hs27 cell lines, with specific antibodies against pentosidine, argpypyrimidine, and N*^ε^*-carboxymethyl-lysine (CML). Pentosidine and argpypyrimidine levels' analysis showed no differences between control and treated cells (data not shown). Likewise, the slot blot analysis revealed no differences in CML protein-bound between control HUES3 and HUES7 and H_2_O_2_ treated cells (Figures 1(c) and 1(d)). On the contrary, somatic cells, Hs27 and HUVEC cell lines, showed a significant increase of CML levels on treated cells by noncytotoxic H_2_O_2_ concentrations, between 8 and 16 *μ*M for Hs27 (Figure 1(e)) and between 16 and 32 *μ*M compared with the lowest H_2_O_2_ concentration (4 *μ*M) for HUVEC (Figure 1(f)).

Immunocytochemical fluorescence staining of the cells showed that CML detection was mainly present in the cytoplasm of HUES lines, and it confirmed no differences between control and treated cells for HUES lines (Supplementary Figures S2 and S3). A very low signal of CML in nontreated somatic cells was slightly increased in terms of fluorescence intensity in somatic H_2_O_2_-treated cells.

### 3.2. Detection of RAGE by Western Blotting and ICC in Treated hES and Somatic Cell Lines

**Figure 2:**
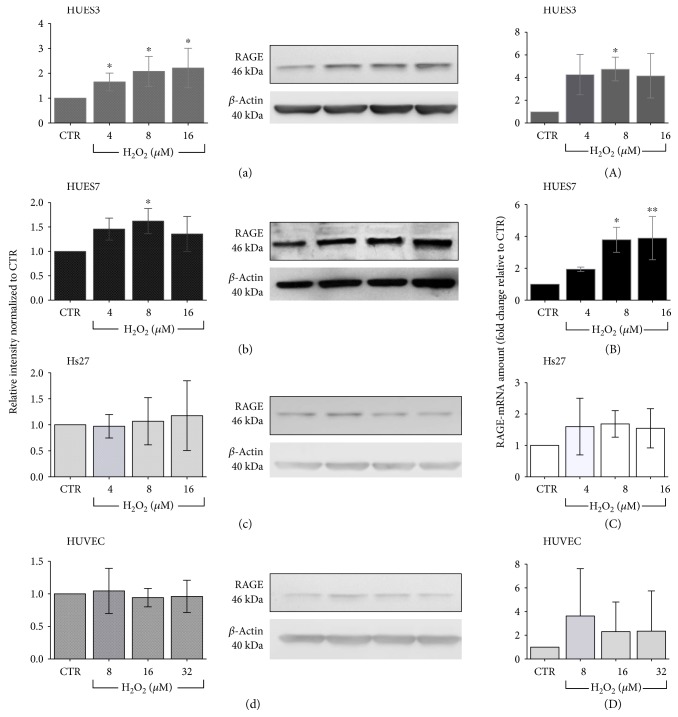
Relative amounts of RAGE protein and mRNA gene expression in hES and somatic cells exposed to H_2_O_2_ for 72 h. (a–d) Relative amount of RAGE in the H_2_O_2_-treated cells compared with the nontreated cells. The *β*-actin serves as a loading control. The intensity of the bands was quantified, and relative values normalized to control condition were shown. The quantification was performed by western blot analysis: mean ± SD, 3 separate replicates per H_2_O_2_ experimental condition. (A–D) RAGE mRNA quantified by real-time RT-PCR in hES and somatic cells treated by noncytotoxic H_2_O_2_ concentrations. mRNA transcripts of RAGE were related to the amount of 18S mRNA molecules. Mean ± SD, 3 separate replicates per H_2_O_2_ experimental condition. Statistically significant differences between groups are indicated as follows: ^∗^*P* ≤ 0.05 and ^∗∗^*P* ≤ 0.01 as established by one-way ANOVA and Tukey's post hoc test.

To clear up the potential effects of oxidative stress at noncytotoxic concentrations on RAGE levels, the global receptor levels were examined by western blot. The RAGE levels were evaluated in three independent experiments. Representative outcomes with an estimation of band intensity and graphical global results are shown in Figure 2. RAGE levels increased significantly after 72 h of H_2_O_2_ exposure in both hESCs, between 4 and 16 *μ*M in HUES3 and between 4 and 8 *μ*M in HUES7 (Figures 2(A) and 2(B)). On the contrary, somatic cells did not show any differences in terms of RAGE levels between control cells and H_2_O_2_ treatment (Figures 2(C) and 2(D)).

Immunocytochemistry confirmed the differences between control and treated cells on hESCs (Supplementary Figures S2 and S3). In somatic cells, the signal detected by immunocytochemistry for CML was very low, so it does not allow to show differences between the controls and the different H_2_O_2_ concentrations.

### 3.3. Detection of mRNA RAGE Expression Levels in Treated Cells with Noncytotoxic H_2_O_2_ Concentrations

To further characterize the levels of RAGE under oxidative stress conditions, we analysed its gene expression by real-time quantitative PCR on undifferentiated and differentiated cell lines treated with noncytotoxic H_2_O_2_ concentrations. HUES3 and HUES7 cell lines showed a significant increase of RAGE mRNA levels in treated cells at 8 *μ*M for HUES3 (Figure 2(A)) and between 4 and 16 *μ*M for HUES7 (Figure 2(B)). In contrast, somatic cells did not show any differences in terms of RAGE gene expression between control cells and H_2_O_2_ treatment (Figures 2(C) and 2(D)). These data coincide with western blot RAGE analysis results.

### 3.4. Neural and Cardiac Differentiation from hES Cells on Feeder-Free Conditions, as a Strategy to Follow-Up CML and RAGE Levels during Development

**Figure 3:**
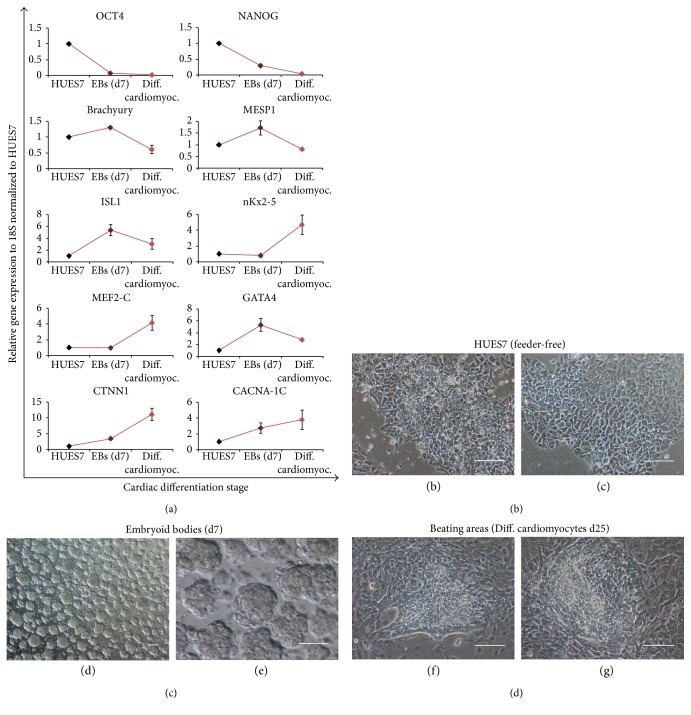
Differentiation of functional hESC-derived CMs. (a) Gene expression patterns during HUES7 cardiomyogenesis. Real-time PCR studies showed that cardiomyocyte differentiation was characterized by a continuous decrease in the expression of pluripotent markers (OCT4 and NANOG) coupled with an initial increase in mesoderm and cardiomesoderm markers (Brachyury and MESP1). This was followed by the expression of a secondary heart field progenitor marker (ISL1), by cardiac-related transcription factor (nKx2-5, MEF2-C, and GATA4), and finally, by a cardiac-specific structural gene (cTNN1) and ionic channels (CACNA-1C). Three qRT-PCR analyses were conducted with each of the 3 independent replicates. Bars depicted the mean ± SD of the relative gene expression at each time point as normalized by baseline values. (b, c) Feeder-free HUES7 cells grown on matrigel. (d, e) Embryoid bodies aggregated from HUES7 cells after 7 days of cardiac differentiation. (f, g). Representation of two areas of contraction on differentiated cardiomyocytes after 25 days of cardiac differentiation. White scale bar = 50 *μ*m.

**Figure 4:**
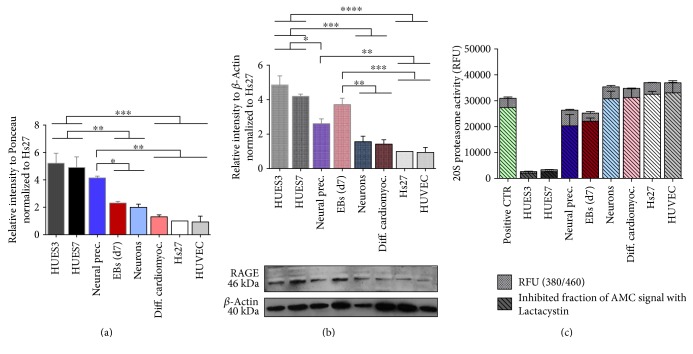
Levels of CML, RAGE, and 20S proteasome activity in protein extracts of hES and somatic cells upon differentiation. (a) Levels of CML in protein extracts of hES (HUES3 and HUES7) and differentiated somatic cells (neural precursors, cardiac embryoids, neurons, cardiomyocytes, Hs27 fibroblast, and HUVEC) upon differentiation. Amount of protein-bound CML related to the protein load (Ponceau S staining) normalized with the Hs27 cell line. Mean ± SD, 3 separate replicates analysed per group. Statistically significant differences between groups are indicated as follows: ^∗^*P* ≤ 0.05, ^∗∗^*P* ≤ 0.01, and ^∗∗∗^*P* ≤ 0.001 as established by one-way ANOVA and Tukey's post hoc test. (b) Levels of RAGE relative to *β*-actin in protein extracts of hES and somatic cells upon differentiation, normalized with the Hs27 cell line. The quantification was performed by western blot analysis: mean ± SD, 3 separate replicates analysed per group. Statistically significant differences between groups are indicated as follows: ^∗^*P* ≤ 0.05, ^∗∗^*P* ≤ 0.01, ^∗∗∗^*P* ≤ 0.001, and ^∗∗∗∗^*P* ≤ 0.0001 as established by one-way ANOVA and Tukey's post hoc test. (c) Proteasome activity: relative fluorescence units measured at 380/460 nm of total sample and inhibited fraction of AMC signal with Lactacystin 20S proteasome inhibitor, mean ± SD, 3 separate replicates analysed per group. Statistically significant differences between groups are indicated as follows: ^∗∗^*P* ≤ 0.01, ^∗∗∗^*P* ≤ 0.001, and ^∗∗∗∗^*P* ≤ 0.0001 as established by one-way ANOVA and Tukey's post hoc test.

To unravel the mechanisms of the differential accumulation of CML and RAGE between embryonic and somatic cells, and in attempt to generate accurate cell models that recapitulate the pathophysiological features of the oxidative stress effects during the embryo development, we induced hES differentiation in two different cell types. We used human neural precursors and neurons derived from hESC in previous work [50]. Since AGEs and RAGE play a central role in the pathogenesis of cardiovascular disease, as previously described [34], we also asked if ES-derived cardiomyocytes have different CML and RAGE basal levels than other differentiated cell lines. To generate functional cardiomyocytes from hES cells line on feeder-free conditions (Figures 3(b) and 3(c)), we follow a protocol through embryoid body (EB) formation (Figures 3(d) and 3(e)) combined with a treatment with a known promoter of cardiac differentiation, the ascorbic acid [51]. The yield of this differentiation protocol varied according to the hESC line, being HUES7 cell line the most successfully differentiated one in comparison with HUES3 and, therefore, the cell line chosen to follow this cardiac differentiation experiments. Spontaneously beating areas of variable size, morphology and pulse rate appeared around 20 days of differentiation (Figures 3(f) and 3(g)). In the same area, we observed cardiomyocytes at different degrees of maturation. We evaluated the temporal gene expression pattern associated with this differentiation process.  Figure 4(a) display the real-time PCR experiments, which show that differentiation was characterized by an initial decrease, at the embryoid body stage, in the expression of undifferentiated pluripotent markers (OCT4 and NANOG). Earliest cardiomyogenesis is linked with a peak in the expression of primitive streak, mesoderm, and cardiomesoderm markers like Brachyury and MESP1 at 7 days of differentiation. After this first event, an increase in the expression of cardiac progenitor markers, such as ISL-1, and cardiac-associated transcription factors nKx2-5, MEF2-C, and GATA4, occurs. Finally, we observed the expression of cardiac-specific structural genes, such as sarcomeric-related proteins (CTNNI) and ion channel proteins (CACNA-1C).

### 3.5. The Levels of CML and RAGE Are Diminished during Differentiation

To determine the basal levels of AGEs and their receptor, at diverse differentiation stages, protein extracts made from undifferentiated and differentiated cells as described above were analysed for the content of CML and RAGE by slot and western blot.

The detection of CML demonstrated that cells that had entered on differentiation displayed lower levels of this AGE than undifferentiated cell lines, being more evident in the cardiac embryoid bodies than in the neural precursors, but decreasing to similar level in cardiomyocytes, neurons, fibroblasts, and endothelial cells (Figure 4(a)). This drop in glycated proteins cannot be explained simply by a dilution of damaged proteins since the undifferentiated embryonic stem cells have a doubling time shorter than their differentiated progenies. In fact, glycated proteins are not eliminated during continuous passages of the undifferentiated and dividing ES cells.

RAGE levels were high in undifferentiated ES cells and decreased upon differentiation (Figure 4(b)). However, cardiac embryoid bodies seemed more similar to their undifferentiated precursors than to the neural precursors, but RAGE level in their differentiated progenies, as in fibroblast and endothelial cells decreased similarly to the CML. Since differentiation process is accompanied by alterations in cellular proteome and cell morphology, we considered the possibility that the reduction in glycated proteins and their receptor could be produced by a reduction in the concentration of specific proteins and not by an elimination of damaged proteins. The lower molecular weight bands present in HUES7 and cardiomyocyte cell protein extracts can be explained by the alternative splicing or cleavage posttranslationally of RAGE [55]. Western blot analysis demonstrated that the overall concentration of protein (*β*-actin) did not change appreciably during differentiation, indicating that the reduction in CML-modified proteins is not a reduction in overall protein modifications. The decrease may result from minor modification of specific proteins and from a lower expression of modifiable, differentially expressed proteins (Figure 4(b)).

Differentiation of hES cells results in an increase in a 20S proteasomal activity.

Previous works have demonstrated the ability of various cell lines to degrade selectively oxidated proteins and that an increase of intracellular proteolysis correlates with mild oxidative stress status [56]. The primary structure responsible for the degradation of oxidatively damaged proteins in the cytosol and nuclei of mammalian cells is the 20S proteasome [39]. To determine whether the reduction in protein damage, in terms of CML and RAGE levels, upon differentiation was concomitant with altered 20S proteasome, its activity was examined using a proteasome activity assay with a fluorescent substrate LLVY-AMC.

This analysis shows that 20S proteasomal activity augments drastically upon differentiation of ES cells (Figure 4(c)). The significant increase of 20S proteasomal activity is shown after 7 days of differentiation at the embryoid body stage of cardiac differentiation, and even more in the differentiated progenies. Differentiation of hES cells support previous results showing a substantial increase in the cells' capacity to degrade oxidatively damaged proteins, coincidently with the decreased levels of CML, and their receptor RAGE [47]. Thus, we confirm that 20S proteasome activity is dynamic and directly activated by oxidants, such as hydrogen peroxide, which induces an enhanced resistance to protein damage and increase the proteolytic removal of damaged proteins [39].

### 3.6. The mRNA RAGE Expression Diminishes during Differentiation

**Figure 5:**
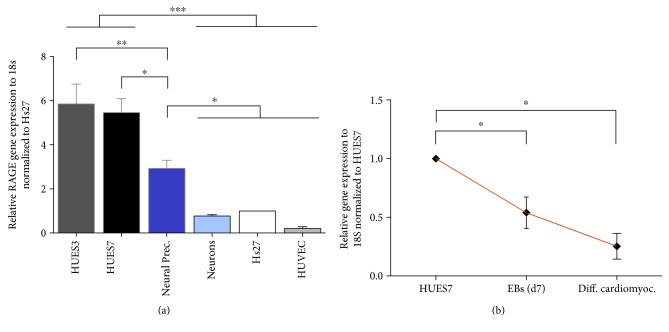
RAGE mRNA quantification by real-time PCR amplification of hES and somatic cells upon differentiation. (a) This study showed a decrease RAGE mRNA in adult neural, Hs27 and HUVEC compared to embryonic cells, 3 independent analyses per group. (b) Moreover, the qPCR analysis showed a decreased number of RAGE transcripts mean ± SD, 3 separate replicates. Statistically significant differences between groups are indicated as follows: ^∗^*P* ≤ 0.05, ^∗∗^*P* ≤ 0.01, and ^∗∗∗^*P* ≤ 0.001 as established by one-way ANOVA and Tukey's post hoc test.

To confirm if the RAGE levels decrease during differentiation in basal conditions, we next examined the mRNA RAGE levels by quantitative real-time PCR in ES cell lines, in their differentiated progenies and on the somatic cell lines Hs27 and HUVEC. Gene expression analysis revealed that RAGE is highly expressed in ES cells and physiologically decreases during differentiation (Figure 5(a)) in agreement with its role during embryogenesis, being crucial among others for neural development and cardiac differentiation (Figure 5(b)).

## 4. Discussion

For decades, scientists have used cell culture to analyse the effect of oxidative stress, providing a wide body of literature with sometime controversial outcomes. During the last years, hypotheses that relate oxidative stress with a wide range of disorders have gained strength. It has been linked with neurodegeneration, diabetes, cardiovascular diseases, reproductive disorders like pre-eclampsia, and aging [26], but the underlying mechanisms are still unknown.

Protein glycation is an indicator of oxidative damage, and the levels of AGEs increase considerably during aging physiological process in animals [57]. Although relevance of AGEs in the pluripotent embryonic stages remains to be clarified, since the accumulation in the embryo is not a consequence of adverse environment, it can be assumed that the low RAGE expression in oocytes and preimplantation embryos prior to blastocyst stage [14] avoid the detrimental effects of AGE-RAGE interaction. Therefore, in our oxidative stress treatment, we incubated both embryonic and somatic cells with different H_2_O_2_ concentrations for 72 h where no effect on cellular viability or proliferation was observed, meanwhile, on the contrary, significant increase in terms of intracellular ROS accumulation and lipid peroxidation has been demonstrated [12].

Several studies proposed that the damaging effect of AGEs is mainly induced by RAGE signalling through a positive feedback increasing inflammatory processes and ROS formation [26]. Moreover, RAGE-ligand interaction leads to an increase in the expression of RAGE itself and its accumulation has been described as an inductor of inflammation in diabetic animal models through NF-k*β* [25]. However, it should be considered that the relevance of AGE-RAGE interactions is still under discussion [58], and the potential problem with these analyses is the nonsimultaneous measure of AGE and RAGE levels in the same experimental context [59]. Our study provides new insights about consequences of an oxidative stress environment during the embryonic preimplantation period by analysing both AGE and RAGE levels in undifferentiated hES and then during their differentiation in somatic cells. The way in which ESCs respond to oxidative stress is still largely unexplored. Although other authors have reported that ESCs are more resistant to oxidative stress than their differentiated fibroblastic progenies [45, 46, 60], we have observed the toxic effect of H_2_O_2_ on cell viability at similar concentrations in both Hs27 and hESCs. However, in terms of AGE and RAGE accumulation, we have seen significant differences between embryonic and somatic cells. Our results confirm that oxidative stress induces an increase of RAGE levels in hESC, in agreement with previous studies in which an adverse maternal preimplantation environment was correlated with an increase in RAGE expression [22]. Curiously, this elevated RAGE mRNA expression does not imply an evident increase of CML; according to the literature, the stability of CML under oxidative stress conditions is probably due to the high basal *plateau* in embryonic pluripotent cells [47]. On the contrary, fibroblasts and endothelial cells exposed to H_2_O_2_ showed a significant increase of CML levels but not in RAGE expression and of protein amount.

It has been proposed that the defensive mechanisms could be more active in cell types whose natural history is associated with acute environmental variations and, particularly, in cells that carry a disproportionate sensitivity and load in maintaining homeostasis for the entire organism in response to a particular stress [61]. In our study, we can hypothesize that HUVEC showed higher resistance to H_2_O_2_ exposure because these cell types in vivo are continuously exposed to shear stress, which has an important impact on cellular metabolism, structure, and function [62], ultimately making them stronger against damage caused by H_2_O_2_ and potentially more active in its elimination. Meanwhile, regarding AGE-RAGE in ES cells, a possible hypothesis is that organisms would invest in a different way in stress-response and protective and corrective mechanisms at the beginning of the developmental process compared to later phases, for the simple reason that the effects of abnormalities are more harmful in the initial moments of the developmental process than at the later stages [44]. In ES cells, AGEs are constitutively present but RAGE expression is not high enough at control conditions to increase the risk of oxidative mechanisms. It is the further increase of CML, induced by H_2_O_2_ treatment, that triggers the activation of RAGE and therefore inducing ROS production. This peculiar regulation of the AGE-RAGE system could provide a mechanistic hypothesis to explain why adverse uterine environments, such as those occurring in diabetic mothers [22], and ART, may negatively affect the embryo quality and induce long-term effects [44].

The source of the observed AGEs in the early embryo pluripotent stem cells is unclear, but there are important considerations to be taken into account: generally, the production of offspring in mammals is coincident with the initial to middle stages of the organism's life cycle, that means that it happens when the overall oxidative damage in the organism is still low [32]. Nonetheless, our results show that early embryonic development steps are accompanied by a drastic reduction of AGE-modified proteins, supporting previous works [47]. AGE formation, especially due to glucose reactions, is a very slow process, and moreover, AGE modifications have previously been shown also in preimplantation mice [47] and rabbit embryos [22] and foetuses of rats [63], it can be hypothesized that the AGE accumulation observed in the inner cell mass of the blastocyst from where ES cells originate derives in part from the progressive build-up of modified proteins starting in germ cells and continuing in oocytes [22] but also could be the result of yet unknown mechanisms that make ES cells have a higher levels that change upon differentiation. To unravel this fact, the analysis of glycation products during neural and cardiac differentiation process has been crucial and allowed us to confirm that the AGE and RAGE levels decrease progressively during organism development.

Several promising studies have shown that elevated levels of RAGE are associated with higher incidence of cardiovascular disease [26] or all-cause mortality in diabetical complications [32]. Indeed, it has been described that blocking the signal transduction of RAGE could be an optimal way to prevent the damage effects of oxidative stress, especially in situations of chronic disease [64, 65]. Pluripotent stem cells have been shown to possess the potential to differentiate spontaneously into CMs, although the efficiency is low, and it depends a lot on each cell line [51]. The aggregation process into EBs mimics the natural developmental course, being possible to generate the physiological tridimensional environment in which the paracrine signals have a key role. Furthermore, with the presence of ascorbic acid in the media, we boosted the cardiac differentiation of pluripotent cells and, as well as, we enhanced their structural characteristics and functional maturation activity [66]. Through this protocol of cardiac differentiation, we studied whether cardiomyocyte-like cells present different basal levels of AGE and RAGE than other somatic cell lines, such as neurons, fibroblast, or endothelial cells, hypothetically turning the cardiomyocytes more sensitive in a hyperglycemic environment. We have not seen any difference in terms of basal CML and RAGE mRNA expression and protein accumulation in comparison with other differentiated cell lines. An explanation of our results could be the still immature state of cardiomyocytes compared to fully differentiated adult cardiac cells [67]; but further experiments should be made for the more accurate determination of the underlying reasons to explain that elevated levels of RAGE are associated with a high incidence of cardiovascular disease. In this line, there are still critical questions that remain to be resolved, so RAGE silencing experiments are planned to a greater understanding of the implications of RAGE on physiological processes.

In this study, we have shown that the low levels of damaged proteins in somatic cells are concurrent with an elevated activity of the 20S proteasome, a fact that has been described essential for the degradation of oxidatively damaged proteins in vivo and in vitro [39]. We also showed that differentiating ES cells from established cell lines cultured in vitro display an efficient removal system of damaged proteins and a simultaneously higher 20S proteasome activity, supporting previous results that demonstrate the elimination of oxidatively damaged proteins occurs during normal embryonic development *in vivo* [47, 48]. Unraveling this mechanism could help to understand the embryonic development process and also help to describe the machinery involved in the aging process, answering to the question of why the differentiated cells invest less in counteracting the gradual accumulation of oxidatively damaged proteins. It has been demonstrated that in vitro 20S can be also controlled by regulators like SDS treatment, opening the structure of 20S and enabling peptide hydrolysis of 20S and therefore its activation, suggesting that all cell types have an equivalent number of 20S particles, because no significant differences in chymotrypsin-like activity were found among the different cells when SDS was added [40]. However, unlike the extensive knowledge acquired over the years respecting the protein degradation process by the 26S proteasome, being described that hESCs have increased levels of the 19S proteasome subunit and a corresponding increased assembly of the 26S/30S proteasome [40], relatively little is known about 20S-mediated proteolysis control.

Alternatively to the 19S particle, the alpha rings of the 20S core can interact, among others, with the cytoplasmic PA28*αβ* (11S) regulator [48]. Moreover, a special conformation of the core proteasome is constituted by replacing the proteolytically catalytic *β*1, *β*2, and *β*5 subunits with *β*1i, *β*2i, and *β*5i subunits, thus forming the immunoproteasome, which has been shown, together with the 20S and PA28*αβ*, to be involved in the adaptation and response to oxidative stress [68]. The analysis of these proteasome structures and regulators is planned for next immediate experiments to better understand their protective role against protein oxidation and during embryogenesis [69]. For the moment, in view of the present results, we can hypothesize that the incorrect functioning of the 20S proteasome leads to the formation of oxidized protein aggregates during cell differentiation.

In conclusion, in this work, we provide a comparative analysis of changes in CML advanced glycation end product and RAGE levels in human embryonic stem cells versus somatic cells upon 72 hours oxidative stress. The cells were exposed to H_2_O_2_ within the noncytotoxic range. Our data reveal that embryonic cells accumulate CML and RAGE under oxidative stress conditions in a different way than somatic cells. Both cell models differ in the basal levels of glycation products, making them a unique model to study the consequence of an oxidative environment on early embryonic cells. Our results confirm the presence of an efficient reduction process of glycated-damaged proteins in hESC, which coincides with a strongly elevated 20S proteasome activity, which has previously been seen in mES cells [47]. The subtle implications of an exposure to oxidative stress during embryonic development are still largely unknown in humans and remain to be elucidated. However, the findings reported here could be a basis to aid the identification of antioxidant treatments for improving embryo culture conditions.

## Supplementary Material

Supplementary figure S1. Quality control of CML antibody specificity. For testing the specificity of the CML antibody human keratin was treated either with methylglyoxal (inducer of argpyrimidine and pentosidine, in lane 1) or glyoxal (inducer of CML, in lane 2). A. Shows the amidoblack staining of the membrane after the blot as loading control. M; molecular weight marker. B. Shows the immunodetection using the anti-CML antibody. As expected only signals in the sample treated with glyoxal were detected demonstrating the specificity of the antibody. Supplementary figure S2. Immunocytochemical analysis of CML and RAGE in control and H2O2-treated HUES3 cells. 24 h post plating cells were treated for 2 hours with increasing concentrations of H2O2. Immunofluorescence staining was performed with an anti-CML (green) and an anti-RAGE (red) antibodies; Hoechst 33342 (blue) was used for nuclei localization. H2O2 conditions: CTR, Control non-treated cells (A), 4 μM (B), 8 μM (C), 16 μM (D). Scale bar = 100 μm. Supplementary figure S3. Immunocytochemical analysis of CML and RAGE in control and H2O2-treated HUES7 cells. 24 h post plating cells were treated for 2 hours with increasing concentrations of H2O2. Immunofluorescence staining was performed with an anti-CML (green) and an anti-RAGE (red) antibodies; Hoechst 33342 (blue) was used for nuclei localization. H2O2 conditions: CTR, Control non-treated cells (A), 4 μM (B), 8 μM (C), 16 μM (D). Scale bar = 100 μm. Supplementary Table T1. Primer sequences used for Real-Time PCR amplification.
