# Supplementary material for: Comparative Analysis of AGE and RAGE Levels in Human Somatic and Embryonic Stem Cells under H2O2-Induced Noncytotoxic Oxidative Stress Conditions
Source: Oxid Med Cell Longev. 2017 Sep 17;2017:4240136. doi: 10.1155/2017/4240136 (PMC5623800; doi:10.1155/2017/4240136)
Supplement: Supplementary file 1 — Supplementary figure S1. Quality control of CML antibody specificity. For testing the specificity of the CML antibody human keratin was treated either with methylglyoxal (inducer of argpyrimidine and pentosidine, in lane 1) or glyoxal (inducer of CML, in lane 2). A. Shows the amidoblack staining of the membrane after the blot as loading control. M; molecular weight marker. B. Shows the immunodetection using the anti-CML antibody. As expected only signals in the sample treated with glyoxal were detected demonstrating the specificity of the antibody. Supplementary figure S2. Immunocytochemical analysis of CML and RAGE in control and H2O2-treated HUES3 cells. 24 h post plating cells were treated for 2 hours with increasing concentrations of H2O2. Immunofluorescence staining was performed with an anti-CML (green) and an anti-RAGE (red) antibodies; Hoechst 33342 (blue) was used for nuclei localization. H2O2 conditions: CTR, Control non-treated cells (A), 4 μM (B), 8 μM (C), 16 μM (D). Scale bar = 100 μm. Supplementary figure S3. Immunocytochemical analysis of CML and RAGE in control and H2O2-treated HUES7 cells. 24 h post plating cells were treated for 2 hours with increasing concentrations of H2O2. Immunofluorescence staining was performed with an anti-CML (green) and an anti-RAGE (red) antibodies; Hoechst 33342 (blue) was used for nuclei localization. H2O2 conditions: CTR, Control non-treated cells (A), 4 μM (B), 8 μM (C), 16 μM (D). Scale bar = 100 μm. Supplementary Table T1. Primer sequences used for Real-Time PCR amplification. [file 4240136.f1.pdf]

Supplementary figure S1. Quality control of CML antibody specificity. For testing the specificity of the CML antibody human keratin was treated either with methylglyoxal (inducer of arg-pyrimidine and pentosidine, in lane 1) or glyoxal (inducer of CML, in lane 2). **A.** Shows the amidoblack staining of the membrane after the blot as loading control. M; molecular weight marker. **B.** Shows the immunodetection using the anti-CML antibody. As expected only signals in the sample treated with glyoxal were detected demonstrating the specificity of the antibody.

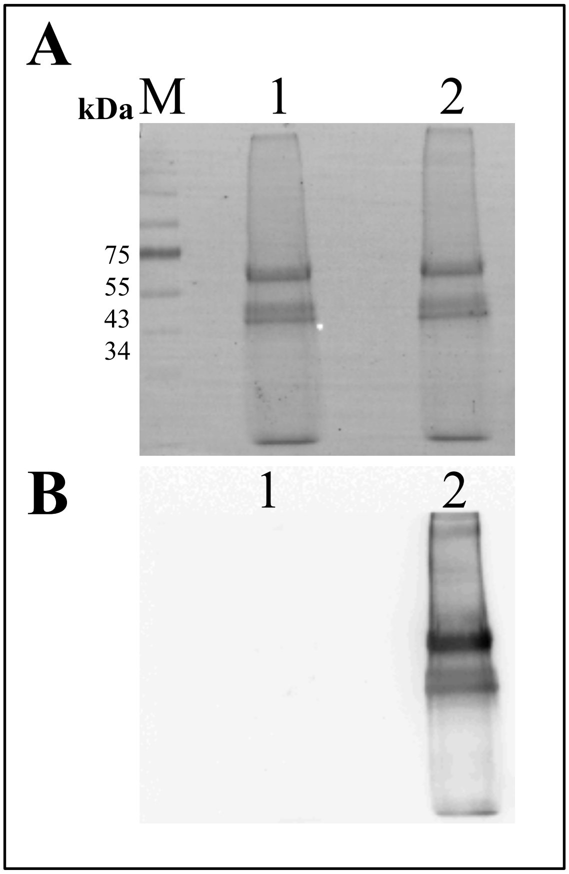

Supplementary figure S2. Immunocytochemical analysis of CML and RAGE in control and H_2O_2-treated HUES3 cells. 24 h post plating cells were treated for 2 hours with increasing concentrations of H_2O_2. Immunofluorescence staining was performed with an anti-CML (green) and an anti-RAGE (red) antibodies; Hoechst 33342 (blue) was used for nuclei localization. H_2O_2 conditions: CTR, Control non-treated cells **(A)**, 4 μM **(B)**, 8 μM **(C)**, 16 μM **(D)**. Scale bar = 100 μm.

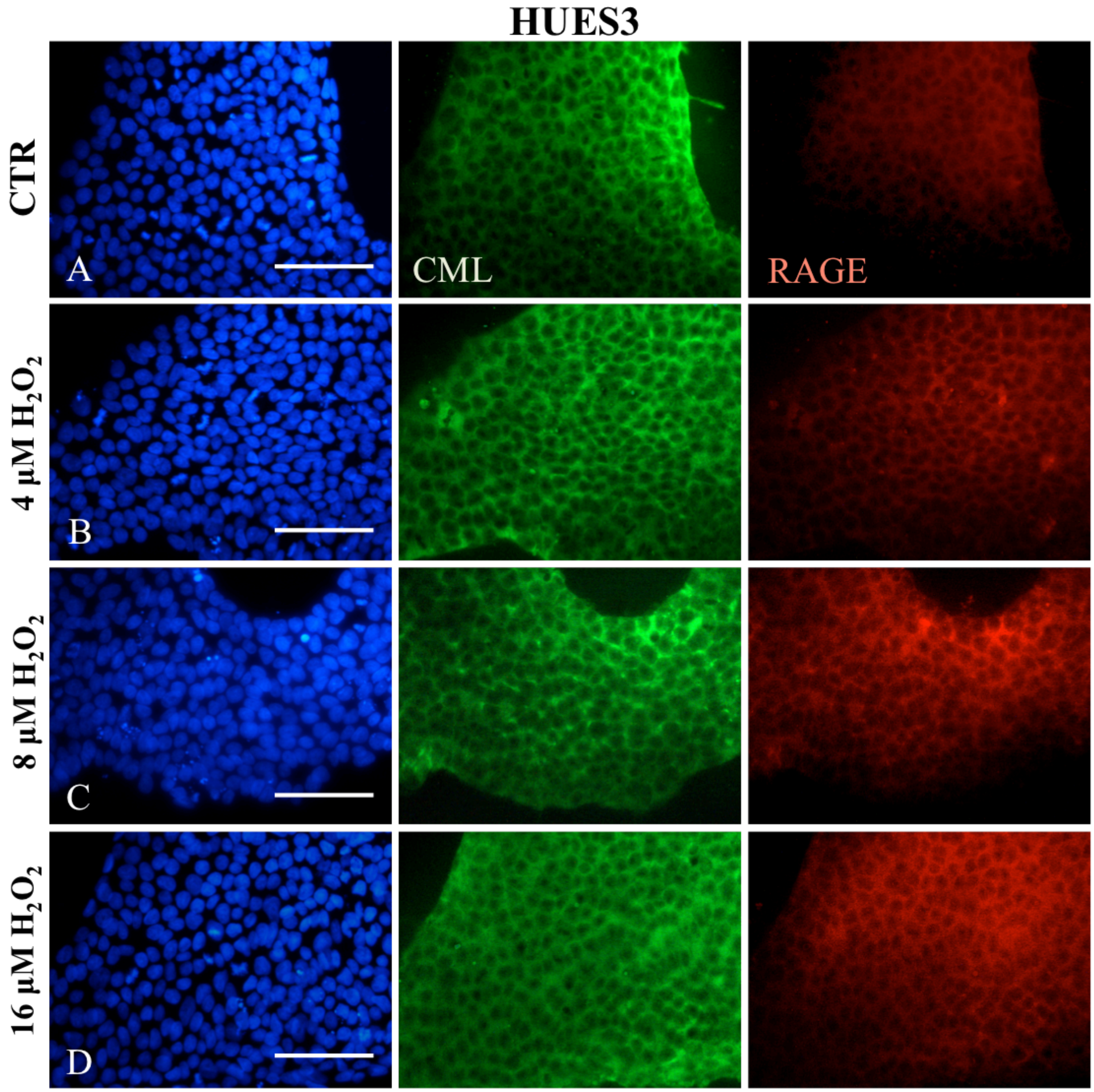

Supplementary figure S3. Immunocytochemical analysis of CML and RAGE in control and H_2O_2-treated HUES7 cells. 24 h post plating cells were treated for 2 hours with increasing concentrations of H_2O_2. Immunofluorescence staining was performed with an anti-CML (green) and an anti-RAGE (red) antibodies; Hoechst 33342 (blue) was used for nuclei localization. H_2O_2 conditions: CTR, Control non-treated cells (**A**), 4 μM (**B**), 8 μM (**C**), 16 μM (**D**). Scale bar = 100 μm.

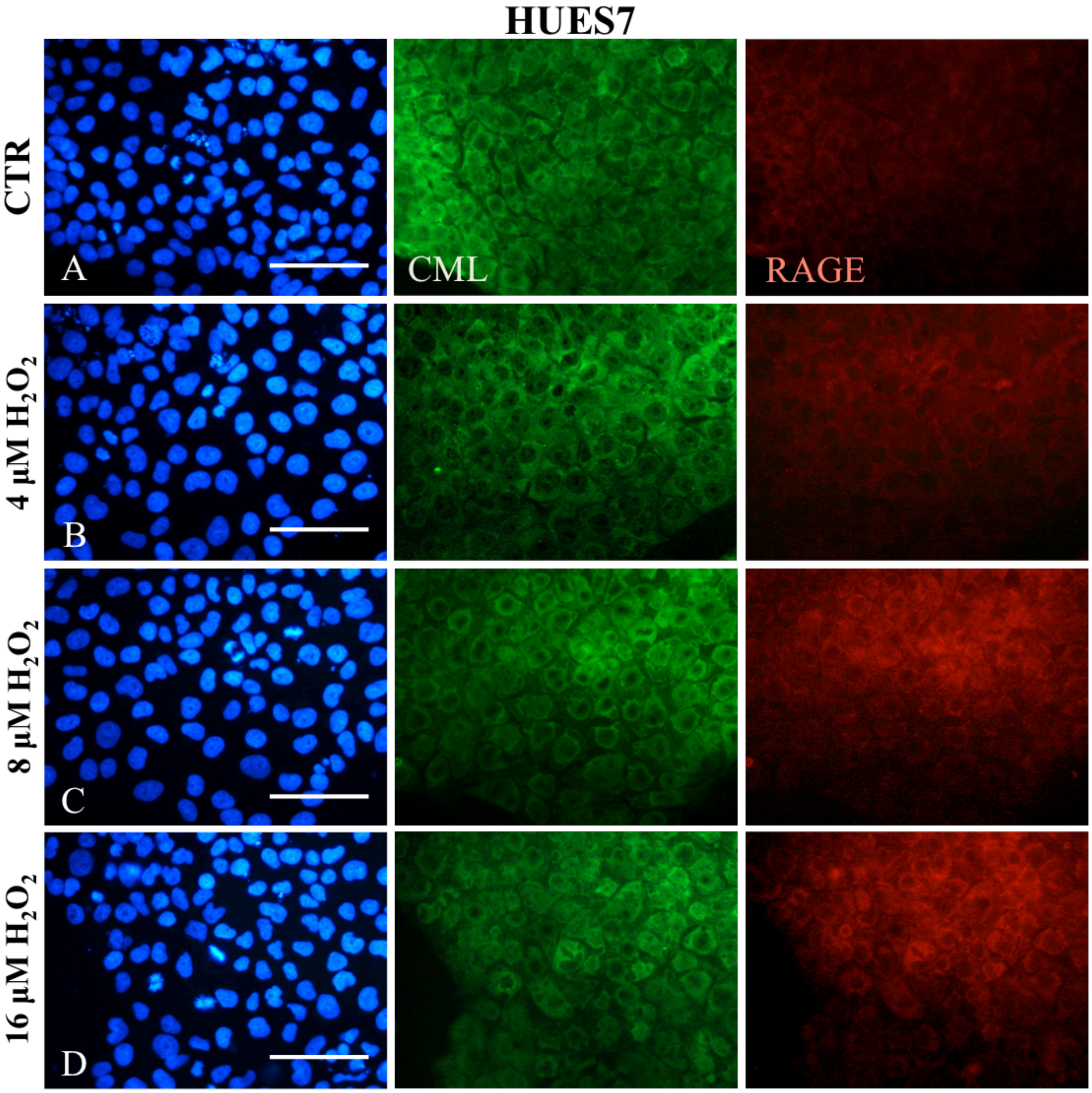

Supplementary Table T1. Primer sequences used for Real-Time PCR amplification

Gene	Forward	Reverse	Annealing T^a [°C]	Product size [bp]
18S	CGCCGCTAGAGGTGAAATTC	TTGGCAAATGCTTTCGCTC	58	62
Brachyury	CAACCTCACTGACGGTGAAAAA	ACAAATTCTGGTGTGCCAAAGTT	58	101
CACNA-1C	AAGGCTACCTGGATTGGATCAC	GCCACGTTTTCGGTGTTGAC	58	136
CTNN1	CCAACTACCGCGCTTATGC	CTCGCTCCAGCTCTTGCTTT	58	120
GATA4	TAGCCCCACAGTTGACACAC	GTCCTGCACAGCCTGCC	58	106
ISL1	GAGGGTTTCTCCGGATTTGG	TCCCATCCCTAACAAAGCATGT	60	101
MEF2-C	TAACTTCTTTTCACTGTTGTGCTCCTT	GCCGCTTTTGGCAAATGTT	58	120
MESP1	CTGCCTGAGGAGCCCAAGT	GCAGTCTGCCAAGGAACCA	58	102
NANOG	TGATTTGTGGGCCTGAAGAAAA	GAGGCATCTCAGCAGAAGACA	56	156
nKx2-5	ACCCTGAGTCCCCTGGATTT	TCACTCATTGCACGCTGCAT	58	125
OCT4	GTGTTCAGCCAAAAGACCATCT	GGCCTGCATGAGGGTTTCT	60	96
RAGE	CTGATCCTCCCACAGAGCC	CAGGACCAGGGAACCTACAG	60	130
